# The Antidepressant Vortioxetine Potently Inhibits Cell Growth in Cultured Human Glioblastoma Cells Expressing 5-HT_1B_ Serotonin Receptors

**DOI:** 10.3390/cells15151323

**Published:** 2026-07-24

**Authors:** Veronica Russo, Miriam Russo, Maria Antonietta Oliva, Marika Alborghetti, Sonia Castaldi, Luisa Di Menna, Giuseppe Battaglia, Ferdinando Nicoletti, Matteo Caridi, Antonietta Arcella

**Affiliations:** 1 IRCCS Neuromed, Via Atinense, 86077 Pozzilli, Italy; veronica.2306@hotmail.it (V.R.); miriamrusso39@gmail.com (M.R.); mariaantonietta.oliva@neuromed.it (M.A.O.); sonia.castaldi@neuromed.it (S.C.); luisa.dimenna@neuromed.it (L.D.M.); giuseppe.battaglia@uniroma1.it (G.B.); ferdinando.nicoletti@uniroma1.it (F.N.); 2 Department of Neuroscience, Mental Health and Sensory Organs, Sapienza University of Rome, 00189 Rome, Italy; marika.alborghetti@uniroma1.it; 3 Department of Physiology and Pharmacology, Sapienza University, 00185 Rome, Italy; matteo.caridi@uniroma1.it

**Keywords:** GBM, Vx, 5-HT_1B_, duloxetine, fluoxetine

## Abstract

**Highlights:**

**What are the main findings?**

- Vortioxetine inhibited glioblastoma (GBM) growth in patient-derived GBM cell lines by suppressing cell pro-liferation at low concentrations (0.1 µM) and inducing apoptosis at higher concentrations (1–5 µM).
- Vortioxetine demonstrated greater anti-GBM efficacy than fluoxetine and duloxetine, with its antitumor effects mediated, at least in part, through activation of the 5-HT1B receptor.

**What is the implication of the main finding?**

- These findings provide further evidence that vortioxetine possesses anti-glioblastoma activity and may be a promising candidate for GBM therapy.
- Given its dual antidepressant and antitumor properties, vortioxetine may offer a therapeutic advantage for patients with GBM who also experience comorbid depression.

**Abstract:**

**Background:** Recent evidence suggests that the antidepressant vortioxetine (Vx) inhibits the growth of glioblastoma (GBM), the most aggressive primary malignant tumor of the CNS. We used five patient-derived GBM cell lines to confirm the anti-GBM effect of Vx and explore its mechanism(s) of action. **Methods:** We performed in silico analysis, PCR, TUNEL assay, colony assay, and proliferation assay. **Results:** Vx potently inhibited GBM cell growth, and showed efficacy at concentrations of 0.1 μM that roughly correspond to therapeutic concentrations of Vx in major depression. Other antidepressants, i.e., fluoxetine and duloxetine, inhibited GBM cell growth only at high concentrations. Computational analysis showed that at least three receptor targets of Vx (5-HT_7_, 5-HT_1D_, and 5-HT_1B_) were expressed at moderate/high levels in GBM. The 5-HT_1B_ receptor transcript was found in all GBM cell lines and was the only detectable Vx target in three of the five cell lines. Blocking 5-HT_1B_ with SB224289 abolished the anti-GBM effect of Vx, even in cells expressing other Vx. **Conclusions:** These findings demonstrate that therapeutic concentrations of Vx inhibit GBM cell proliferation and suggest that this action may be mediated, at least in part, by 5-HT_1B_ receptor activation.

## 1. Introduction

Serotonin (5-HT) has been implicated in the pathophysiology of different types of cancer, such as melanoma and colorectal, hepatocellular, gastric, breast, pancreatic, prostate, lung, ovarian, bladder, biliary tract, and neuroendocrine cancers [1]. The action of 5-HT is mediated by different classes of receptors, including 5-HT_1A_, 5-HT_1B_, 5-HT_1D_, 5-HT_1E_, 5-HT_1F_, and 5-HT_5_ receptors, which are coupled to G_i/o_ proteins; 5-HT_2A_, 5-HT_2B_, and 5-HT_2C_ receptors, which are coupled to G_q/11_ proteins; 5-HT_3_ receptors, which are ligand gated cation channels; and 5-HT_4_, 5-HT_6_, and 5-HT_7_ receptors, which are coupled to G_s_ proteins [2]. 5-HT receptors display pleiotropic functions in cancer cells, regulating cell proliferation, survival, and migration in a context-dependent fashion. For example, 5-HT_1A_ and 5-HT_1B_ receptor antagonists have been proposed as putative therapeutic targets in prostate and colorectal cancer, whereas inhibition of 5-HT_1A_, 5-HT_2A_, 5-HT_2B_, 5-HT_4_, and 5-HT_6_ was shown to restrain proliferation of cholangiocarcinoma cells [1]. Until recently (see below), a role for 5-HT in the pathophysiology of malignant gliomas has been inferred by the evidence that selective serotonin reuptake inhibitors (SSRIs), such as fluoxetine and escitalopram, caused cell death in glioma cell lines, or reduced xenografted glioblastoma (GBM) growth in nude mice [3,4]. However, no association was found between the use of SSRIs and overall survival of patients with GBM [5].

GBM is the most common adult-type primary malignant brain tumor, characterized by an isocitrate dehydrogenase (IDH) wild-type phenotype and a poor prognosis. The standard of care is maximal safe surgical removal followed by radiotherapy and chemotherapy. Temozolomide (TMZ) is the primary chemotherapeutic agent owing to its efficacy as a DNA alkylating agent and its ability to cross the blood–brain barrier. Resistance of glioma cells to TMZ develops as a result of O^6^-methyguanine-DNA methyltransferase (MGMT) expression or other mechanisms (reviewed by Weller et al., 2024) [6]. In spite of the increased understanding of the molecular pathogenesis and the development of new targeted therapies, the treatment of GBM remains suboptimal, with a median survival rate < 15 months [6]. High-throughput screening of different classes of neuroactive compounds led to the identification of vortioxetine (Vx) as the most effective anti-GBM drug [7]. In orthotopic human xenograft GBM mouse models, Vx significantly enhanced survival, synergized with standard-of-care DNA alkylating agents, and reduced brain tumor size in vivo [7]. Vx-containing microspheres have shown anti-cancer activity in 2D monolayer or 3D spheroid GBM cells derived from patients [8]. Vx is a multimodal antidepressant with great efficacy in improving cognitive dysfunction, anhedonia, and emotional blunting in patients affected by major depressive disorder (MDD) [9,10]. Vx is an inhibitor of the high affinity serotonin transporter (SERT), a full agonist of 5-HT_1A_ serotonin receptors, a partial agonist of 5-HT_1B_ receptors, and an antagonist of 5-HT_1D_, 5-HT_3_, and 5-HT_7_ receptors [11]. It is unknown which of these targets mediates the anti-GBM activity of Vx. Gene and protein network analysis and molecular docking simulation revealed five intracellular protein targets for Vx, including AKT1, a serine/threonine protein kinase, which signals downstream of phosphatidylinositol-3-kinase (PI3K) [12]. 5-HT_1D_ receptor blocking may contribute to the anti-GBM activity of Vx because the receptor is linked to the PI3K pathway, and its overexpression enhances proliferation, migration, and resistance to temozolomide in GBM cells [13]. However, direct evidence that links the 5-HT_1D_ receptor or any other serotonin receptor to the suppressing activity of Vx on GBM growth is lacking. We examined the action of Vx on five GBM cell lines derived from patient biopsies addressing the following questions: (I) is the anti-cancer activity of Vx uniform and concentration-dependent in all cell lines? (II) Does Vx affect cell proliferation or cause cell death depending on the concentrations? (II) Does Vx action correlate with the expression of one or more drug targets? (IV) If so, is the action of Vx affected by pharmacological manipulation of the selected target? In addition, we compared Vx with two other antidepressants, i.e., the selective serotonin reuptake inhibitor (SSRI), fluoxetine (Fx), and the serotonin–noradrenaline reuptake inhibitor (SSNRI), duloxetine (Dx). The in vitro study was preceded by a computational analysis of SERT and serotonin receptor expression in GBMs.

## 2. Materials and Methods

- Data sources and analysis

The study included two publicly available RNA-seq datasets, TCGA and CPTAC. Raw RNA-seq and clinical data were retrieved from cBioPortal (https://www.cbioportal.org/, accessed on 10 March 2025). All samples with available transcriptomic data were included in the analysis. The TCGA dataset comprised 166 glioblastoma multiforme samples, of which 153 were primary tumors and 13 were recurrent tumors. The CPTAC dataset contained 99 primary GBM samples. Both datasets were used to evaluate the expression levels of the genes of interest. The TCGA cohort was further employed for survival analyses and for the reconstruction of gene co-expression networks and interactomes. Expression values were log_2_-transformed using the formula log_2_(x + 1) in order to stabilize variance and reduce right-skewness of the distributions. A predefined panel of genes of interest, based on the Vx targets, was selected, including serotonin receptor genes (HTR_3A_, HTR_3B_, HTR_3C_, HTR_3D_, HTR_3E_, HTR_1A_, HTR_1B_, HTR_1D_, and HTR_7_), the serotonin transporter gene (SLC6A4), cyclooxygenase genes (PTGS1, PTGS2), and the housekeeping gene β-ACT. The expression matrix was subset to retain only these genes for downstream analyses. All analyses were performed using RStudio (R version 4.3.1, “Beagle Scouts”, R Foundation for Statistical Computing, Vienna, Austria) running on macOS (x86_64-apple-darwin20). The following R packages were used for data import, preprocessing, statistical analyses, survival analysis, visualization, and functional enrichment: readxl, survival, ggplot2, survminer, WGCNA, clusterProfiler, org.Hs.eg.db, AnnotationDbi, KEGGREST, Cairo, openxlsx, and reshape2. The corresponding references could be generated using the R citation () function.

- Descriptive statistics of gene expression and data visualization

For each selected gene, descriptive statistics (minimum, 25th percentile, mean, median, 75th percentile, and maximum) were computed; kernel density of these genes estimates of log_2_-transformed expression values across samples were computed and visualized. Density curves were plotted with enhanced graphical parameters and semi-transparent area filling to facilitate visual assessment of expression distribution and variability.

- Survival analysis

The association between gene expression and overall survival was evaluated using Cox proportional hazards regression models. For each gene, univariate Cox models were fitted using log_2_-transformed expression values as continuous predictors and overall survival time as the outcome. Given the established prognostic relevance of clinical variables, such as age at diagnosis and Karnofsky Performance Status (KPS), analyses were adjusted for age alone, KPS alone, and for both variables simultaneously. Hazard ratios, 95% confidence intervals, Wald statistics, and *p*-values were estimated for each gene. Multiple testing correction was applied using the Benjamini–Hochberg false discovery rate (FDR) method.

- Kaplan–Meier survival analysis and log-rank testing

For each gene of interest, survival differences were further explored using Kaplan–Meier analysis with Mantel–Cox (log-rank) tests. Log_2_-transformed gene expression values were used to stratify patients into putative prognostic groups according to both median-based dichotomization (high vs. low expression) and percentile-based categorization (low: <25th percentile; intermediate: 25th–75th percentile; and high: >75th percentile). Overall survival was defined in months, and event status was coded as death versus censoring. Survival curves were estimated using the Kaplan–Meier method and compared between expression-defined groups using the log-rank test. For each gene, plots were generated including 95% confidence intervals and numbers at risk. This approach allowed a non-parametric, visually intuitive assessment of the prognostic impact of single-gene expression levels, complementing the Cox regression analyses and providing an additional evaluation of survival separation across clinically relevant expression strata.

- Weighted Gene Co-expression Network Analysis (WGCNA)

Gene Co-expression Network Analysis was performed using the WGCNA framework on log_2_-transformed TCGA RNA-seq expression data. After quality control using “goodSamplesGenes”, the analysis was restricted to the 8000 genes with the highest variance in order to reduce noise and improve computational feasibility. Expression data were transposed to obtain samples as rows and genes as columns. Hierarchical clustering was then used to inspect potential outlier samples. The soft-thresholding power was selected using a data-driven criterion based on the scale-free topology fit index and mean connectivity. Candidate powers were evaluated using “pickSoftThreshold”. The first power reaching R^2^ ≥ 0.80 was 5, which also corresponded to the knee-point of the R^2^ curve. The first power reaching the more stringent R^2^ ≥ 0.90 threshold was 7. The final soft-thresholding power was selected according to a saturation criterion, defined as the first power with R^2^ ≥ 0.80 and a subsequent R^2^ increase ≤ 0.03. Based on this criterion, “softPower = 7” was selected. At this power, the scale-free topology fit index was 0.915, the subsequent R^2^ gain was 0.013, and the mean connectivity was 7.3. The soft-thresholding power selection curve, including the scale-free topology fit index and mean connectivity across the tested powers, is reported in Figure S1. This choice was therefore considered an appropriate compromise between approximate scale-free topology and preservation of sufficient network connectivity. Using the selected soft-thresholding power, an adjacency matrix was generated and transformed into a Topological Overlap Matrix. Genes were hierarchically clustered based on TOM dissimilarity, and modules were detected using the dynamic tree cut algorithm with “deepSplit = 2”, “pamRespectsDendro = FALSE”, and a minimum module size of 20 genes. Module eigengenes were computed and clustered to assess inter-module similarity. Highly similar modules were merged using an eigengene dissimilarity threshold of 0.15, corresponding to a module eigengene correlation of 0.85.

- Module membership and gene co-expression network of HTR_1B_, HTR_1D_, and HTR_7_

Genes were assigned to WGCNA modules, and the modules containing HTR_1B_, HTR_1D_, and HTR_7_ were identified. Gene co-expression network (GCN) analysis of up-regulated genes was performed by computing Spearman correlation matrices. Correlation coefficients and FDR-adjusted *p*-values were obtained. For intramodular correlation analyses, different correlation thresholds were applied according to the strength of the association observed for each receptor. For HTR_7_, genes were defined as strongly associated using a stringent cutoff (ρ > 0.6, FDR < 0.05). Because correlations for HTR_1D_ were overall weaker, the threshold was relaxed to ρ > 0.4 (FDR < 0.05). For HTR_1B_, which showed even lower correlation coefficients, a further reduced cutoff of ρ > 0.35 (FDR < 0.05) was adopted. This adaptive approach allowed the identification of biologically meaningful co-expressed genes while accounting for receptor-specific differences in correlation strength.

- Functional Enrichment Analysis of HTR_7_-, HTR_1D_-, and HTR_1B_-Associated Gene Sets

Functional enrichment analysis was performed on gene sets defined as belonging to the same WGCNA module as each receptor (HTR7, HTR1D, and HTR1B) and showing significant correlation with the corresponding receptor. As the background universe, we used the genes retained for WGCNA network construction, namely the 8000 genes with the highest variance after quality control. Gene Ontology (GO) Biological Process and KEGG pathway enrichment analyses were carried out with the clusterProfiler package, applying the Benjamini–Hochberg method for multiple testing correction (adjusted *p*-value < 0.05). Enriched terms were visualized using dot plots, and the results were exported for downstream interpretation.

- Cell Culture

Primary human GBM cells (DNA p15, CLp14, CGp15, COGIp14, and NULUp12) (*p* refers to the number of passages from the establishment of the first culture) were cultured in vitro from brain biopsies of Neuromed neurosurgery patients who gave informed consent to participate in this study. The use of primary cell lines as a model for GBM heterogeneity was approved by the ethics committee on 27 February 2020 and registered on ClinicalTrials.gov with the identification number NCT04180046. GBM cell lines, used in the following experiments, were characterized as previously reported [14]. In detail, primary GBM cells were cultured in Dulbecco’s Modified Eagle’s Medium (DMEM) supplemented with 10% Fetal Bovine Serum (FBS), 2 mmol/L-glutamine, 100 IU/mL Penicillin, and 100 g/mL Streptomycin at 37 °C, 5% CO_2_, and 95% humidity. Table 1 shows the main characteristics of primary cells from grade II gliomas and grade IV GBM samples derived from Neuromed patients, according to the 2016 WHO classification [14]. We used the MycoAllert Mycoplasma Detection Kit (Lonza, Basel, Switzerland) for the detection of mycoplasma contamination.

- Proliferation assay

All patient-derived GBM cells were seeded in 48-well plates at 1 × 10^4^ cells per well in DMEM supplemented with 10% FBS and cultured at 37 °C in an atmosphere containing 5% CO_2_. Primary GBM lines growing in adhesion to the plate were treated daily with Vx (Sigma Aldrich, St. Louis, MO, USA), Dx, or Fx (Merck, KGaA, Darmstadt, Germany) in amounts of 0.1, 1, 5, 10, and 20 μM for 24, 48, and 72 h. After washing with DPBS 1X, cells were detached with 0.25% trypsin, resuspended in 300 μL of medium, and then counted in a Burker chamber. Cell number counts were plotted in graph curves using GraphPad Prism version 8. Statistical analysis of the curves was performed using two-way ANOVA with multiple comparisons. Statistical significance was set at **** *p* < 0.0001. Furthermore, SB224289 HYDROCLORIDE 0.1–1 μM (5-HT_1B_ Antagonist) (TOCRIS, Bristol, UK) was administrated 20 min before Vx treatment (0.1, 1 μM). For controls, DMSO 0.1% was used.

- PCR

Total RNA was extracted from DNA, CL, CG, COGI, and NULU cells by using the Direct-Zol RNA microprep kit (ZYMO RESEARCH, Irvine, CA, USA) while cDNA was synthesized using the SuperScript™ III First-Strand Synthesis System (Invitrogen, Carlsbad, CA, USA). cDNA was then amplified by using the following predesigned gene-specific primers (Metabion, Planegg, Bavaria, Germany) with PCR. All samples were analyzed in triplicate and β-actin (β-ACT) was used as a reference gene. The sequences of the primers used in the study are reported in Table 2.

- Clonogenic Assay

The colony formation assay was performed by plating 1 × 10^3^ cells per well on 6-well plates for 24 h. CG and NULU cells were treated with Vx 5 and 10 μM and DMSO 0.1% (Sigma Aldrich, St. Louis, MO, USA) as the control for 72 h. Subsequently, the medium was replaced every 3 days for 15 days. For the evaluation of the clonogenic potential of the CG and NULU primary GBM lines and the determination of the number of colonies formed under the different treatment conditions, the cells were fixed with a 4% paraformaldehyde solution for 20 min, washed with DPBS 1X, and stained with 0.05% crystal violet for 30 min. The stained colonies were counted under an optical microscope at 40× magnification (scale bar = 50 μm).

- DNA Fragmentation’s Detection by TUNEL Assay

NULU, CG, COGI, DNA, and CL cells were plated in 8-well chamber slides (1 × 10^5^ cells/well) in DMEM 0.5% FBS for 48 h and then treated with 0.1 and 5 µM for 48 h. After treatment, cells were fixed in 4% methanol-free formaldehyde solution, pH 7.4, for 25 min at 4 °C and washed twice with DPBS 1X. DNA fragmentation was detected by Terminal deoxynucleotidyl transferase (TdT) dUTP Nick-End Labeling (TUNEL) assay with the commercial kit DeadEnd Fluorometric TUNEL System (Promega, Madison, WI, USA), following the manufacturer’s instructions. Cells were counterstained with DAPI mounting medium (Vectashield, Vector Labs, Newark, California, CA, USA) and analyzed with a fluorescence microscope (Axiophot 2 Zeiss, Oberkochen, Germany) at 40× magnification.

- Protein Extraction and Western Blot Analysis

Proteins were extracted from primary GBM cells of patients and mouse tissues with Triton X-100 lysis buffer (10 mM Tris-HCl, 1 mM EDTA, 150 mM NaCl, 1% Triton X-100, 1 mM NaF, 1 mM Na4P2O7, 1 mM Na3VO4, and 1× protease inhibitors). After quantitation by the Bradford assay, 20 µg of total lysate were separated by sodium dodecyl sulfate-polyacrylamide gel electrophoresis (SDS-PAGE) and transferred onto Immuno-Blot^®^ PVDF Membrane. The membrane was incubated with 4% BSA solution for 2 h at room temperature and then overnight at 4 °C with rabbit polyclonal anti-5-HT1B (dilution 1:1000, Novus Biotechnologies, Centennial, CO, USA). The signals were detected by a digital scanner and quantified with Image Lab 6.1 Software (Bio-Rad Laboratories, Inc., Hercules, CA, USA). For protein normalization, the membrane was incubated with mouse monoclonal antibody anti-GAPDH (dilution 1:2500, Abcam, Cambridge, UK).

- Measurement of serotonin (5-HT) levels

Cell culture medium samples were stored at −80 °C until analysis. One hundred and eighty µl of each sample were mixed with 20 µL of 1 N perchloric acid and then centrifuged at 14,000× *g* for 20 min. Supernatants were transferred into loading vials for the quantification of serotonin 5-HT. The analysis was performed by an Agilent 1220 Infinity II liquid chromatography system (Agilent Technologies, Santa Clara, CA, USA), with molecules separated on an analytical C-18 reverse-phase column (Ultrasphere ODS 5 µm, 80 Ǻ pore, 250 × 4.6 mm) (Avantor, Radnor Township, PA, USA) and a DECADE Elite electrochemical detector (Antec Scientific, Hoorn, The Netherlands). The holding potentials were set at +350 and −350 mV for the detection of 5-HT. The mobile phase consisted of 80 mM sodium phosphate, 40 mM citric acid, 0.4 mM ethylenediaminetetraacetic acid, 3 mM 1-heptansulfonic acid, and 15% methanol brought to pH 2.75 with phosphoric acid (run under isocratic conditions, at 1 mL/min). The concentrations of 5-HT were quantified by calibration curves generated from serial dilutions of commercially available standards.

- Measurement of cAMP formation in cells

Cells were incubated in Krebs–Henseleit buffer (equilibrated with 95% O_2_/5% CO_2_, pH 7.4) for 35–45 min at 37 °C under constant oxygenation to allow metabolic recovery. 3-isobutyl-1-methylxanthine (IBMX), 0.5 mM, was added, and then 15 min later cells were challenged with vortioxetine (0.1, 1 or 5 μM) and/or SB224289 (1 μM) with or without forskolin (10 μM), which was added 2 min after the drugs. The incubation was continued for 20 min. The reaction was stopped by adding ice-cold 0.4 N HClO_4_. The samples were then rapidly placed on ice and left at −20 °C overnight. On the day of the experiment, the samples were sonicated for 10–15 s and then added to 2 N K_2_CO_3_ and centrifuged at low speed. An aliquot of the supernatant was diluted and used for cyclic AMP determination. Cyclic AMP formation was quantified using a commercially available enzyme-linked immunosorbent assay (ELISA) kit (Tema Ricerca, Castenaso, BO, Italy). IBMX, forskolin, vortioxetine, and SB224289 were purchased from Sigma-Aldrich (St. Louis, MO, USA).

## 3. Results

### 3.1. Computational Analysis of VX Targets in GBM

#### 3.1.1. Vx Targets Are Expressed in GBM and Show Potential Prognostic Impact

**Figure 1:**
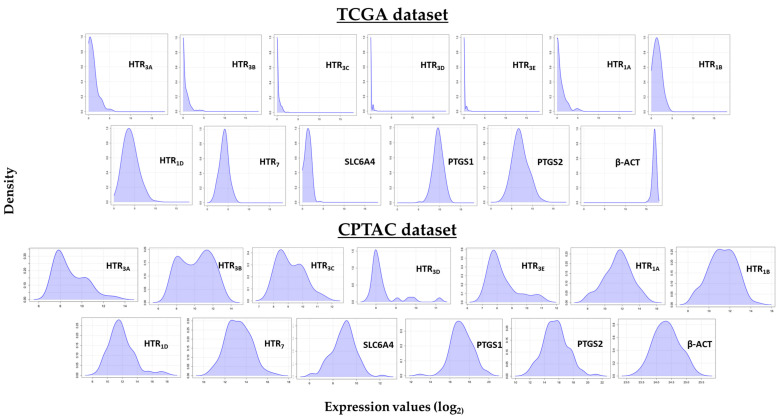
The graph shows a density curve representing the distribution of LOG expression values for each gene. On the *X*-axis, the graph shows expression measures, and on the *Y*-axis, it shows the frequency with which these values occur. PTGS1, PTGS2, and 5-HT_7_ show the highest expression levels.

Standard statistical analyses and density plots were generated to assess Vx target gene (Vx gene) expression based on RNAseq. PTGS1, PTGS2, and 5-HT_7_ were the most highly expressed genes across both datasets, followed by 5-HT_1D_ and 5-HT_1B_. 5-HT_3_-related genes exhibited the lowest expression levels (Figure 1, Tables S1 and S2).

**Figure 2:**
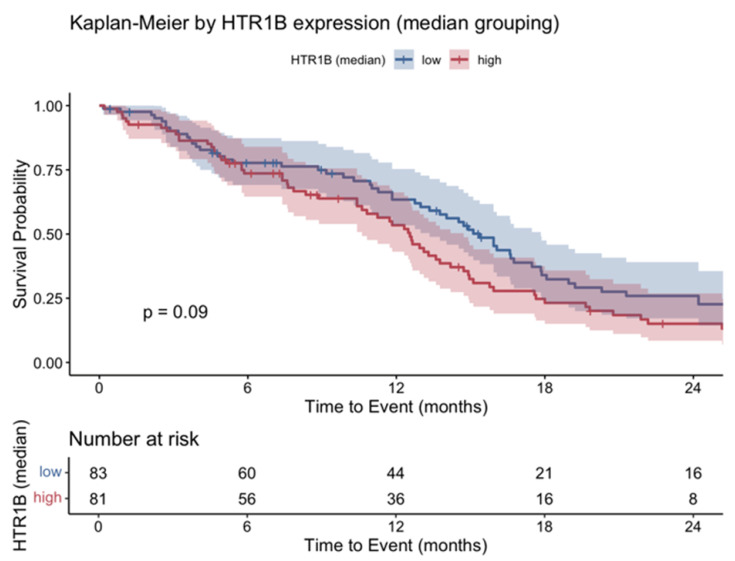
Kaplan–Meier survival curve displaying overall survival stratified by median 5-HT_1B_ expression (high vs. low). The *X*-axis represents time to event in months (0–24), and the *Y*-axis shows survival probability. The plot includes confidence intervals, a log-rank test *p*-value, and a risk table indicating the number of subjects at risk at 6-month intervals.

**Figure 3:**
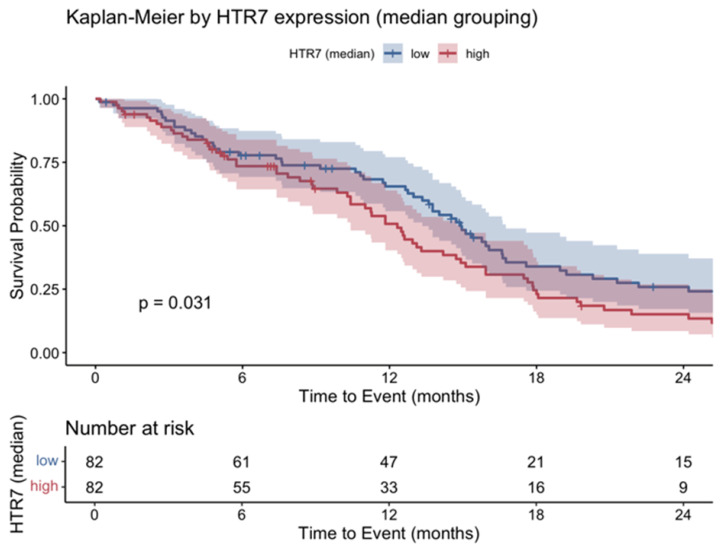
Kaplan–Meier survival curve displaying overall survival stratified by median 5-HT_7_ expression (high vs. low). The *X*-axis represents time to event in months (0–24), and the *Y*-axis shows survival probability. The plot includes confidence intervals, a log-rank test *p*-value, and a risk table indicating the number of subjects at risk at 6-month intervals.

Univariate Cox proportional hazards models were fitted using gene expression levels as continuous variables and overall survival as the endpoint, with adjustment for important clinical prognostic factors (age at diagnosis and Karnofsky Performance Status—KPS). The results are shown in Tables S3–S5. In these adjusted models, high levels of 5-HT_1B_ showed a statistically significant association with patient outcome when stratifying for KPS alone and for the combined effect of KPS and age, indicating its potential prognostic relevance in GBM (HR = 1.28, *p* = 0.035). 5-HT_7_ expression showed a trend toward an adverse prognostic impact (HR = 1.19, *p* = 0.095). Stratification of Kaplan–Meier curves based on median and percentile expression levels further supported a putative worse prognostic effect associated with high expression of both 5-HT_1B_ and 5-HT_7_ (Figure 2 and Figure 3; Figures S2 and S3).

#### 3.1.2. Interactome Reconstruction Shows a Tight Association Between Serotonin Receptors and an Immune Signature in GBM

We investigated the interactomes of 5-HT_1B_, 5-HT_1D_, and 5-HT_7_ in GBM by performing Weighted Gene Co-expression Network Analysis (WGCNA) to identify the main co-expression modules associated with each receptor and to reconstruct their corresponding co-expression networks. To infer the potential biological roles of these targets, functional enrichment analyses were subsequently performed, focusing on Gene Ontology Biological Processes and KEGG pathways.

While the 5-HT_1B_-associated module contained too few genes to allow robust functional inference, well-defined interactomes were obtained for 5-HT_1D_ and, in particular, for 5-HT_7_. Several enriched pathways in these networks, especially within the 5-HT_7_ interactome, were related to immune functions, including “leukocyte-mediated immunity”, “regulation of cytokine production”, “leukocyte activation”, and “leukocyte transendothelial migration”. Considering the strong immune component of the GBM microenvironment, the potential role of serotonergic receptors in GBM may be linked both to immune cell infiltration and to a direct functional involvement of tumor cells displaying an immune-like transcriptional program. Consistently, many of the enriched GO and KEGG terms were associated with cellular activation, proliferation, and differentiation processes, such as leukocyte differentiation, T cell activation, mononuclear cell differentiation”, etc. (Figures S4 and S5).

### 3.2. Study of Vx in Human GBM Cell Culture

**Figure 4:**
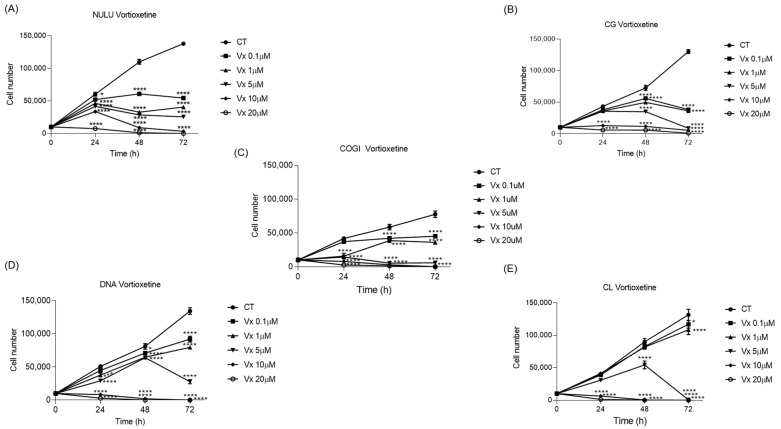
Cell growth inhibition was assessed after 24, 48, and 72 h of daily treatment with Vx at concentrations of 0.1, 1, 5, 10, and 20 μM. Growth curves were generated for NULU (**A**), CG (**B**), COGI (**C**), DNA (**D**), and CL (**E**) based on cell counts at the indicated time points (*n* = 3). Statistical analysis was performed using two-way ANOVA with Tukey’s multiple comparisons test using GraphPad Prism 8 software. * *p* < 0.05; *** <0.001, and **** < 0.0001 vs. CT; F_time (3, 168)_ = 438.9, F_treatments (5, 168)_ = 822.1, F_interaction (15, 168)_ = 167.5, *p* = 0.0001 for NULU cells; F_time (3, 167)_ = 255.7, F_treatments (5, 167)_ = 447.9, F_interaction (15, 167)_ = 118.7, *p* = 0.0001 for CG cells; F_time (3, 168)_ = 81.25, F_treatments (5, 168)_ = 287.2, F_interaction (15, 168)_ = 45.71, *p* = 0.0001 for COGI cells; F_time (3, 167)_ = 498.9, F_treatments (5, 167)_ = 579.6, F_interaction (15, 167)_ = 122.5, *p* = 0.0001 for DNA cells; and F_time (3, 168)_ = 296.5, F_treatments (5, 168)_ = 345.9, F_interaction (15, 168)_ = 85.55, *p* = 0.0001 for CL cells.

We used primary cultures prepared from human GBM biopsies to examine the effect of Vx on GBM cell growth. Five GBM cultures (called NULU, CL, COGI, CG, and DNA) were treated daily with increasing concentrations of Vx (0.1, 1, 5, 10, and 20 μM) for 72 h (Figure 4). Vx inhibited GBM cell growth in all cultures with greater potency in NULU and CG, in which 0.1 μM of Vx reduced cell growth by more than 50%. At 20 μM, Vx abolished cell growth in all cultures (Figure 4).

**Figure 5:**
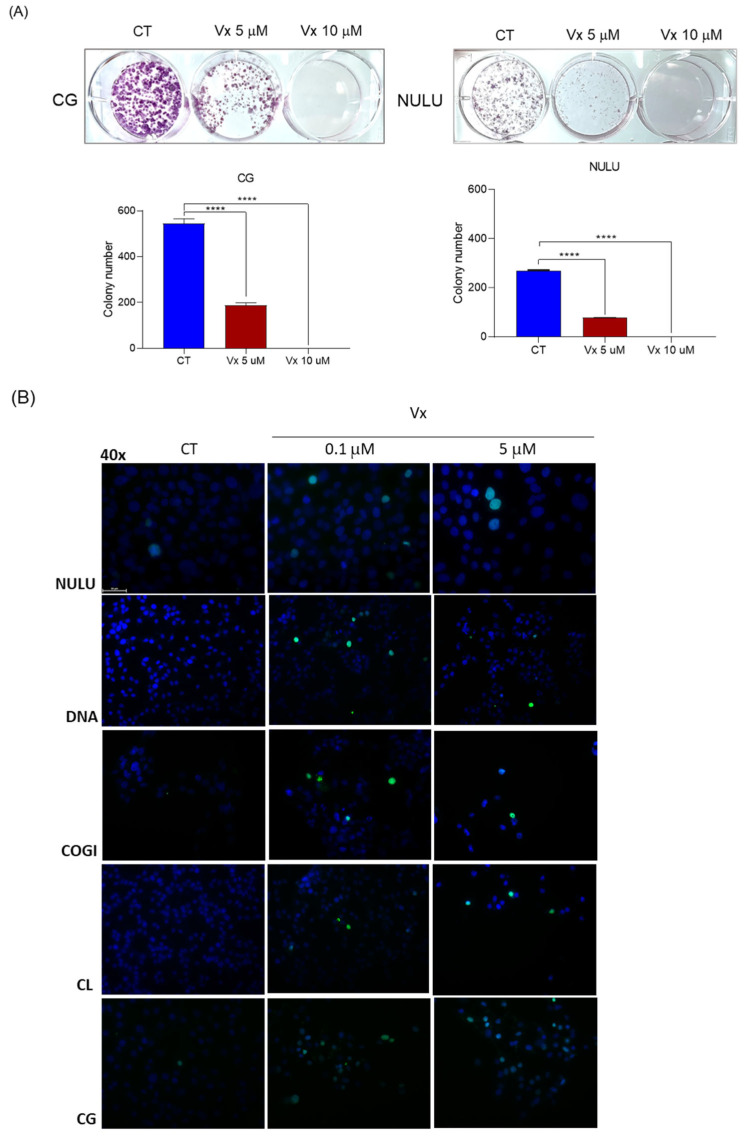
Effect of Vx on the clonogenic potential of CG and NULU cells. (**A**) Representative images and corresponding quantification of clonogenic assays performed in CG and NULU cell lines. Cells were treated with Vx (5 and 10 μM) and the treatment-containing medium was refreshed every 3 days over a 15-day period. DMSO-treated cells were used as controls (*n* = 3). Statistical analysis was performed using one-way ANOVA and Tukey’s multiple comparisons test using GraphPad Prism 8 software; **** *p* value < 0.0001 vs. CT; F_(2, 6)_ = 1263 for CG cells and F_(2, 6)_ = 14,845 for NULU cells. (**B**) Representative images of TUNEL assay results in NULU, DNA, COGI, CL, and CG cells, which were treated with Vx (0.1 and 5 μM) for 48 h, compared with untreated controls (CT) (*n* = 3). Apoptotic cells are shown in green, and DAPI was used to stain the nuclei. Images were acquired at 40× magnification; scale bar = 50 μm.

Inhibition of GBM cell growth by Vx was confirmed by the colony formation assay performed with 5 and 10 μM of Vx in CG and NULU cells (Figure 5A). Subsequently, apoptosis was detected in NULU, DNA, COGI and CL cells 48 h after Vx treatment at final concentrations of 0.1 and 5 μM. As shown in Figure 5B, the proportion of apoptotic cells increased in a dose-dependent manner.

**Figure 6:**
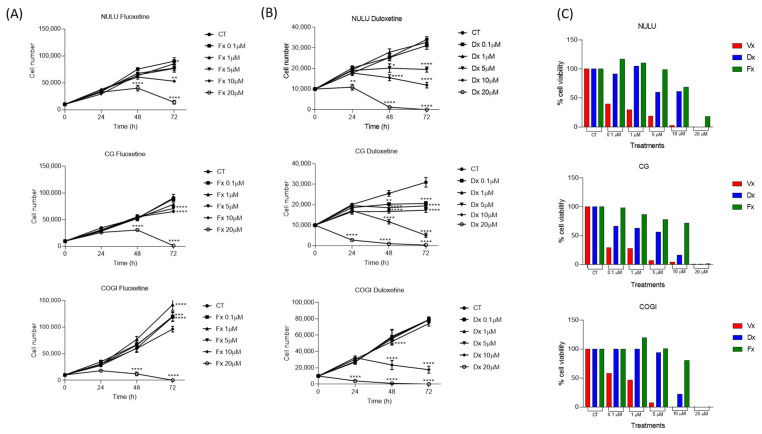
Proliferation of GBM primary cells (NULU, CG, and COGI) was evaluated after 24, 48, and 72 h of daily treatment with (**A**) Fx and (**B**) Dx at concentrations of 0.1, 1, 5, 10, and 20 μM. Cell counts were performed at each indicated time point (*n* = 3). Statistical analysis was performed using two-way ANOVA with Tukey’s multiple comparisons test using GraphPad Prism 8 software. (**A**) * *p* value < 0.05, ** < 0.01, *** < 0.001 and **** < 0.0001 vs. CT; F_time (3, 168)_ = 329.0, F_treatments (5, 168)_ = 32.27, F_interaction (15, 168)_ = 14.90, *p* = 0.0001 for NULU cells; F_time (3, 164)_ = 471.8, F_treatments (5, 164)_ = 63.07, F_interaction (15, 164)_ = 30.92, *p* = 0.0001 for CG cells; F_time (3, 163)_ = 328.4, F_treatments (5, 163)_ = 66.44, F_interaction (15, 163)_ = 22.56, *p* = 0.0001 for COGI cells. (**B**) * *p* value < 0.01, ** < 0.001, and **** < 0.0001 vs. CT; F_time (3, 168)_ = 98.10, F_treatments (5, 168)_ = 113.6, F _interaction (15, 168)_ = 26.76, *p* = 0.0001 for NULU cells; F_time (3, 168)_ = 56.00, F_treatments (5, 168)_ = 184.00, F _interaction (15, 168)_ = 31.08, *p* = 0.0001 for CG cells; F_time (3, 165)_ = 354.9, F_treatments (5, 165)_ = 170.7, F_interaction (15, 165)_ = 44.88, *p* = 0.0001 for COGI cells. (**C**) Comparative graphs illustrate cell viability following treatment with Vx (red columns), Fx (green columns), and Dx (blue columns) across the same concentration range (0.1–20 μM).

NULU, CG, and COGI cultures were also challenged with equimolar concentrations of Fx and Dx for comparative purposes. Fx reduced cell growth in the three cultures only at the highest concentration (20 µM) (Figure 6A), whereas Dx reduced cell growth at concentrations > 5 µM in NULU, at all concentrations in CG, and only at concentrations of 20 µM in COGI (Figure 6B). Comparative analysis showed a greater potency of Vx in inhibiting GBM cell growth compared to Fx and Dx (Figure 6C).

**Figure 7:**
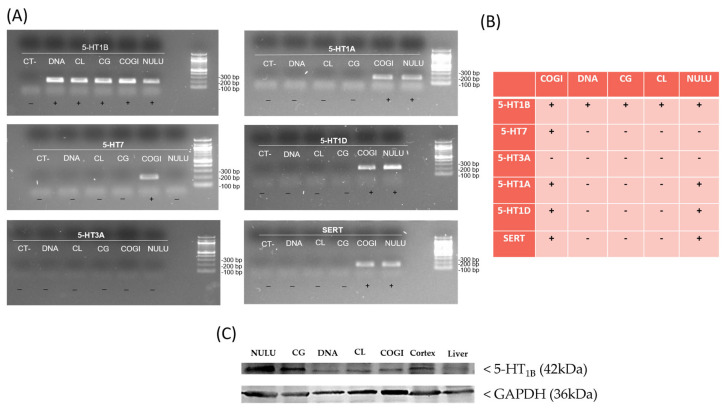
Expression of Vx targets in GBM cell lines. PCR analysis of 5-HT_1B_, 5-HT_7_, 5-HT_3A_, 5-HT_1A_, and 5-HT_1D_ receptors, and SERT is shown in (**A**). The absence or presence of the different transcripts is summarized in (**B**). (**C**) Western blot analysis of 5-HT_1B_ receptor protein levels in GBM cell lines is shown in (**C**), where the mouse cerebral cortex was used as a positive control.

**Figure 8:**
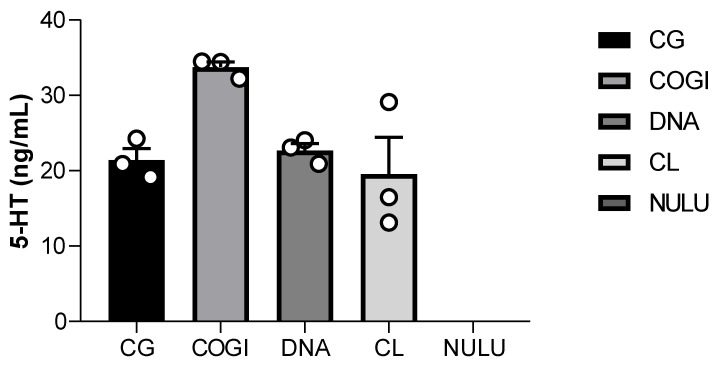
Extracellular 5-HT levels in GBM cell lines. Values are means + S.E.M. of 3 samples per group (*n* = 3).

We examined the transcripts of Vx targets (i.e., SERT and 5-HT_1A_, 5-HT_1B_, 5-HT_1D_, 5-HT_3A_, and 5-HT_7_ receptors) in all cultures. The transcript of 5-HT_1B_ was detected in all cultures and was the only transcript detected in CL, COGI, and DNA cultures, whereas the transcript of 5-HT_3A_ receptors was below the detection levels in all cultures. All transcripts were detected in COGI and NULU culture, with the exception of 5-HT_3A_ in COGI and 5-HT_3A_ and 5-HT_7_ in NULU (Figure 7A,B). 5-HT_1B_ protein levels were also detected in all cell lines by immunoblot analysis (Figure 7C). 5-HT was detected in the medium of all cell lines with the exception of NULU, in which 5-HT was below the detection levels (Figure 8).

**Figure 9:**
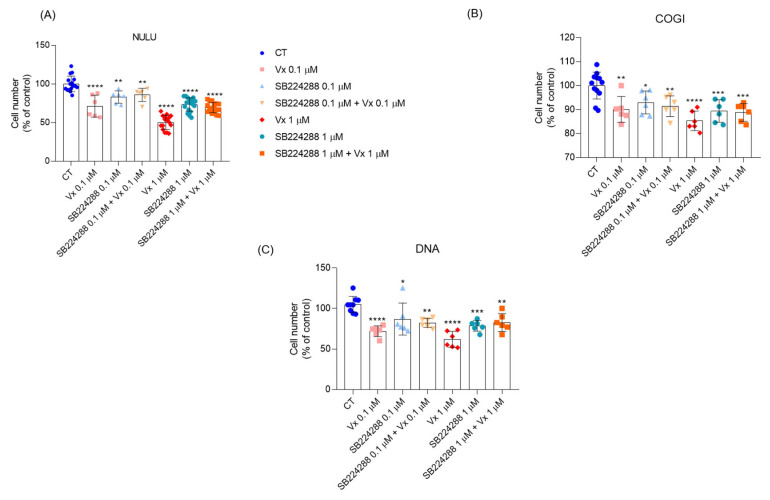
Pharmacological blockade of 5-HT_1B_ receptors abolishes the antiproliferative action of Vx in GBM cell lines. Proliferation assays of NULU (**A**), COGI (**B**), and DNA (**C**) cell lines treated with Vx (0.1 or 1 μM) and the 5-HT_1B_ receptor antagonist (SB224289, 0.1 or 1 μM), administered alone or in combination. Cell counts were performed at a specific time point (48 h) (*n* = 3). Statistical analysis was performed using one-way ANOVA followed by Dunnett’s multiple comparisons test using GraphPad Prism 8 software. Statistical significance is indicated as follows: * *p* value < 0.05, ** < 0.01, *** < 0.001, and **** < 0.0001 vs. CT; F _interaction (6, 79)_ = 43.86, *p* = 0.0001 for NULU cells.

Because Vx inhibited cell growth in all cultures, we examined the possibility that the anti-GBM activity of Vx could be mediated by the activation of 5-HT_1B_ receptors. We challenged NULU, COGI, and DNA cell lines with 0.1 and 1 µM Vx in the absence or presence of the selective 5-HT_1B_ receptor antagonist, SB224289 (0.1 and 1 µM). SB224289 slightly reduced cell growth on its own, but abolished the inhibitory action of Vx in all three cell lines (Figure 9A–C).

Knowing that 5-HT_1B_ receptors are coupled to G_i/o_ proteins, we measured cAMP formation in two cell lines challenged with Vx and/or SB224288 in the absence or presence of forskolin, which stimulates adenylyl cyclase activity. Vx reduced forskolin-stimulated cAMP formation only at concentrations of 1 or 10 μM, but was ineffective at 0.1 μM. SB224288 also reduced forskolin-stimulated cAMP formation, and occluded the action of high concentrations of Vx (Figures S6 and S7).

## 4. Discussion

Our findings fully confirm the potent anti-GBM activity of Vx [7,8,12,13] and provide new insights into the underlying mechanism(s). Vx was effective in reducing GBM cell proliferation in our cultures at concentrations of 0.1 μM, which roughly corresponds to plasma concentrations in response to therapeutic doses of Vx in patients affected by major depressive disorder (Cmax values: 8–33 ng/mL corresponding to 0.02–0.11 μM) [15,16]. Vx at concentrations > 0.1 μM caused apoptotic cell death in all GBM cell lines.

Other antidepressants, including SSRIs and SNRIs, were shown to reduce GBM cell growth in previous studies. For example, the SSRI fluoxetine caused apoptotic death of cultured GBM cells by enhancing Ca^2+^ influx through AMPA-gated ion channels and activating the CHOP pathway or through other mechanisms, and showed synergistic activity with TMZ by suppressing MGMT expression. These effects were always seen at concentrations > 10 μM [3,17,18,19,20,21]. The SNRI duloxetine reduced glioma growth in in vivo studies, but did not affect glioma cell proliferation or viability at concentrations < 10 μM [22]. In our GBM cell lines, both duloxetine and fluoxetine were able to reduce cell growth/viability, but only at high concentrations, and they were considerably less potent than Vx.

Transcriptomic, proteomic, and phosphoproteomic profiling of glioma cells showed that AP1 transcription factors, BTG1, and related intracellular pathways, such as the ERK pathway, endoplasmic reticulum stress pathway, and DNA damage responses, were up-regulated, whereas oncogenic tyrosine kinase receptors were down-regulated after treatment with Vx [7]. Another study revealed five core targets of Vx, i.e., AKT1, type-1 hypoxia inducible factor (HF-1), cadherin-E, the p105 subunit of NFκB, and prostaglandin endoperoxide synthase 2, and concluded that the anti-GBM action of Vx was mediated by modulation of multiple processes, including cell metabolism, cell migration, inflammation, apoptotic death, and Rap1 signaling and HIF-1 [12]. We wondered whether one or more membrane protein targets of Vx (SERT, and 5-HT_1A_, 5-HT_1B_, 5-HT_1D_, 5-HT_3_, and 5-HT_7_ receptors) are involved in the anti-cancer action of Vx. Addressing this question is highly relevant from a therapeutic standpoint because membrane transporters and receptors can be targeted by selective ligands. Zhang et al. showed that overexpression of 5-HT_1D_ enhanced proliferation and migration of GBM cells and cell resistance to TMZ by inducing MGMT expression [13]. Our computational analysis showed a high/moderate expression of 5-HT_7_, 5-HT_1D_, and 5-HT_1B_ receptor mRNAs in two GBM datasets, supporting the hypothesis that the 5-HT_1D_ receptor can be one of the targets for the action of Vx in GBM cells [13]. Unexpectedly, however, 5-HT_1D_ and 5-HT_7_ receptor mRNA levels were detectable only in two cell lines (NULU and COGI), whereas all cell lines expressed the transcript of 5-HT_1B_ receptors. The loss of 5-HT_1D_, 5-HT_7_, and other Vx targets in four of our GBM cell lines might be a consequence of the culture conditions. Vx was also effective in reducing cell proliferation in cell lines in which only the transcript encoding the 5-HT_1B_ was detectable. Vx behaves as a partial agonist of 5-HT_1B_ receptors (see Section 1 and References therein), and, therefore, we used a selective 5-HT_1B_ receptor antagonist (compound SB224289) to assess the role of 5-HT_1B_ receptors in the action of Vx. SB224289 slightly reduced GBM cell growth on its own but abolished the antiproliferative action of Vx in the three cell lines. This suggests that activation of 5-HT_1B_ receptors is likely involved in the antiproliferative action of Vx in NULU, COGI, and DNA cell lines. We cannot exclude that other Vx targets are involved, particularly in NULU and COGI cell lines in which the transcript of all Vx targets was detected with the exception of the 5-HT_3_ receptor. The 5-HT_7_ receptor is a potential candidate because our computational analysis showed high levels of 5-HT_7_ mRNA in GMB. However, Vx acts as a 5-HT_7_ receptor antagonist and this mechanism might contribute to the action of Vx only if the receptor is activated by endogenous 5-HT. This is unlikely to occur in NULU cell lines, in which 5-HT levels were undetectable in the cell medium, unless the 5-HT_7_ receptor is constitutively active and Vx behaves as an inverse agonist. This hypothesis warrants further investigation.

5-HT_1B_ receptors are coupled to Gi proteins, and their activation inhibits cAMP formation [23]. This mechanism may account for the anti-GBM action of Vx because cAMP drives glioma cell proliferation [24,25,26]. In two cell lines, micromolar concentrations of Vx reduced forskolin-stimulated cAMP formation, but the drug was inactive at concentrations of 0.1 μM, which displayed anti-proliferative effects. Whether or not inhibition of cAMP formation was mediated by 5-HT_1B_ receptors remains to be determined because the antagonist SB224288 also inhibited cAMP formation in our cell lines. Activation of any receptor coupled to G_i/o_ proteins may engage multiple transduction pathways through the βγ subunits of the G protein, and some of these pathways, such as the MAPkinase and phosphtidylinositl-3-kinase pathways, critically regulate cancer cell growth and survival. Thus, further studies are needed to establish whether Vx restrains GBM cell proliferation by activating 5-HT_1B_ receptors and through which mechanism. Additional genetic approaches (e.g., successful knockdown or knockout studies) will be required in future work to establish the contribution of the 5-HT_1B_ receptor conclusively.

Another potential target of Vx, which we have not investigated here, is type-2 cycloxygenase (COX2). Prostaglandin-E2 (PGE2), the major downstream product of COX2, may promote glioma growth [27], as suggested by the evidence that the percentage of COX2-positive cells is higher in high-grade gliomas, and correlates with a poor prognosis [28], and elevated COX2/PGE2 correlates with reduced survival after radiotherapy [29,30], as reviewed by Dean and Hooks, 2023 [31]. Vx was found to potently inhibit COX2 activity in isolated human monocytes [32]. Inhibition of cAMP formation resulting from 5-HT_1B_ receptor activation and inhibition of COX2 might be linked to the same pathway of anti-GBM growth by Vx because cAMP can induce COX2 by activating the cAMP-response element binding protein (CREBP) [33].

## 5. Conclusions

In conclusion, our findings strengthen the evidence that Vx retrains GBM cell growth, demonstrate that this action is displayed at therapeutic concentrations, and suggest that, under our experimental conditions, activation of 5-HT_1B_ receptors may contribute to one of the underlying mechanisms. A clinical trial with Vx for newly diagnosed GBM is on-going (NCT07284628). Regardless of the expected findings, Vx might represent a valuable drug for the treatment of depression in patients with GBM. In addition, Vx has an optimal profile of safety and tolerability, with the exception of nausea, which can be present in 10–29% of patients [33] and can be handled with slow dose titration [34,35]. However, this raises some concerns because both radiotherapy and TMZ treatment may cause nausea in GBM patients.

## Figures and Tables

**Table 1:** Clinical characteristics of the Neuromed patients and the primary cell cultures derived from their biopsies.

ID	GENDER	AGE	LOCALIZATION	DIAGNOSIS	IDH1 Status	MGMT	VIMENTIN	GFAP	ATRX WT	Ki67	P53
COGI	M	62	Frontal	GBM	Wild-type	Methylated	+	+	+	50%	Low
CL	M	62	Frontal	GBM	Wild-type	Unmethylated	+	+	+	40%	Low
NULU	F	35	Temporoparietal	GBM	Wild-type	Unmethylated	+	Focal expression	+	20%	Low
CG	F	55	Temporoinsular	GBM	Wild-type	Methylated	+	Focal expression	+	4%	High
DNA	M	74	Temporal	GBM	Wild-type	Unmethylated	+	Focal expression	+	15%	Low

**Table 2:** Primer sequences for serotonin receptor genes, the serotonin transporter gene, and housekeeping genes.

	Primers	Sequence
1	5-HT_1A__FOR	5′-TCCACCTTTGGAGCTTTCTAC-3′
	5-HT_1A__REV	5′-TCTCCATTCACACTCTTCTTGG-3′
2	5-HT_1B__FOR	5′-TCATGATCGCGCTGGTGTGG-3′
	5-HT_1B__REV	5′-TAGAGGGCGATGAGGAGCAG-3′
3	5-HT_1D__FOR	5′-ACTCACCACCATCTTACTCAC-3′
	5-HT_1D__REV	5′-AGCAATGACACAGAGATGCA-3′
4	5-HT_3A__FOR	5′-TCAATGAGTTCGTGGATGTGG-3′
	5-HT_3A__REV	5′-TTGATGTCCTGGATGGTGTG-3′
5	5-HT_7__FOR	5′-TGATCAGCATTGACAGGTACC-3′
	5-HT_7__REV	5′-ATCGTATAGCCAAAGTCCTGG-3′
6	SERT_FOR	5′-CAGCGTGTGAAGATGGAGAAG-3′
	SERT_REV	5′-TGGGATAGAGTGCCGTGTGT-3′
7	β-ACT_FOR	5′-GCCAGACAGCACTGTGT-3′
	β-ACT_REV	5′-GTGCGTGACATTAAGGAG-3′

## Data Availability

The data that support the findings of this study are available from the corresponding author upon reasonable request.

## Supplementary Materials

The following supporting information can be downloaded at: https://www.mdpi.com/article/10.3390/cells15151323/s1, Figure S1: Soft-thresholding power selection for WGCNA network construction; Figure S2: Kaplan–Meier survival curve displaying overall survival stratified by HTR1B percentile-based categorization; Figure S3: Kaplan–Meier survival curve displaying overall survival stratified by HTR7 percentile-based categorization; Figure S4: Functional enrichment of HTR7-associated genes; Figure S5: Functional enrichment of HTR1D-associated genes; Figures S6 and S7: Measurement of cAMP formation in cells; Tables S1 and S2: Summary statistics(min, 25th percentile, mean, media 75th percentile, and max of log2-trasformed RNA-seq expression levels from TGCA dataset; Tables S3–S5: Multivariable Cox proportional hazards analyses.

## Abbreviations

The following abbreviations are used in this manuscript:

5-HT_1A_	5-hydroxytryptamine Receptor 1A
5-HT_1B_	5-hydroxytryptamine Receptor 1B
5-HT_1D_	5-hydroxytryptamine Receptor 1D
5-HT_3_	5-hydroxytryptamine Type 3 Receptor
5-HT_7_	5-hydroxytryptamine Type 7 Receptor
5HT	Serotonin
ACTB	Actin Beta
AKT1	Ak Strain Transforming 1
AMPA	α-amino-3-hydroxy-5-methyl-4-isoxazolepropionic acid
AP1	Activator Protein-1
BTG1	B-cell Translocation Gene 1
cAMP	Cyclic Adenosine Monophosphate
cDNA	Complementary Deoxyribonucleic Acid
CHOP	Cyclophosphamide, Hydroxydaunorubicin (doxorubicin/Adriamycin), Oncovin (vincristine), and Prednisone.
CNS	Central Nervous System
COX2	Type-2 cycloxygenase
CPTAC	Clinical Proteomic Tumor Analysis Consortium
CREBP	cAMP-response Element Binding Protein
DMEM	Dulbecco’s Modified Eagle’s Medium
DNA	Deoxyribonucleic Acid
DPBS	Dulbecco’s Phosphate Buffered Saline
Dx	Duloxetine
FBS	Fetal Bovine Serum
FDR	False Discovery Rate
Fx	Fluoxetine
GBM	Glioblastoma
GCN	Gene Co-expression Network
GO	Gene Ontology
HF-1	Hypoxia-Inducible Factor 1
IDH	Isocitrate Dehydrogenase
KEGG	Kyoto Encyclopedia of Genes and Genomes
KEGGREST	Client-side REST access to the Kyoto Encyclopedia of Genes and Genomes
KPS	Karnofsky Performance Status
MDD	Major Depression Disorder
MGMT	O6-methylguanine-DNA methyltransferase
mRNA	Messenger Ribonucleic Acid
NFκB	Nuclear Factor Kappa-light-chain-enhancer of Activated B Cells
PCR	Polymerase Chain Reaction
PGE2	Prostaglandin-E2
PI3K	Phosphatidylinositol 3-kinase
PTGS1	Prostaglandin Endoperoxide Synthase 1
PTGS2	Prostaglandin Endoperoxide Synthase 2
RAP-1	Ras-related Protein 1
RNA	Ribonucleic Acid
SERT	Serotonin Transporter
SLC6A4	Solute Carrier Family 6 Member 4
SSNRI	Serotonin–Noradrenaline Reuptake Inhibitor
SSRI	Selective Serotonin Reuptake Inhibitor
TCGA	The Cancer Genome Atlas
TMZ	Temozolomide
TOM	Topological Overlap Matrix
TUNEL	Terminal Deoxynucleotidyl Transferase (TdT) dUTP Nick-End Labeling
Vx	Vortioxetine
WGCNA	Weighted Gene Co-expression Network Analysis
WHO	World Health Organization
